# Heterogeneous Visible-Thermal and Visible-Infrared Face Recognition Using Cross-Modality Discriminator Network and Unit-Class Loss

**DOI:** 10.1155/2022/4623368

**Published:** 2022-03-11

**Authors:** Usman Cheema, Mobeen Ahmad, Dongil Han, Seungbin Moon

**Affiliations:** Department of Computer Engineering, Sejong University, Seoul, Republic of Korea

## Abstract

Heterogeneous face recognition (HFR) aims to match face images across different imaging domains such as visible-to-infrared and visible-to-thermal. Recently, the increasing utility of nonvisible imaging has increased the application prospects of HFR in areas such as biometrics, security, and surveillance. HFR is a challenging variate of face recognition due to the differences between different imaging domains. While the current research has proposed image preprocessing, feature extraction, or common subspace projection for HFR, the optimization of these multi-stage methods is a challenging task as each step needs to be optimized separately and the performance error accumulates over each stage. In this paper, we propose a unified end-to-end Cross-Modality Discriminator Network (CMDN) for HFR. The proposed network uses a Deep Relational Discriminator module to learn deep feature relations for cross-domain face matching. Simultaneously, the CMDN is used to extract modality-independent embedding vectors for face images. The CMDN parameters are optimized using a novel Unit-Class Loss that shows higher stability and accuracy over other popular metric-learning loss functions. The experimental results on five popular HFR datasets demonstrate that the proposed method achieves significant improvement over the existing state-of-the-art methods.

## 1. Introduction

The applications of facial recognition (FR) [1] systems have increased exponentially with the advent of deep convolutional neural networks. Currently, automated FR is being used in personal devices, public surveillance, access control, security, marketing, and other applications. While the performance of FR algorithms has achieved near-human accuracy for frontal images, the performance is limited in scenarios involving extreme variations in illumination, expression, pose, presentation attacks, and disguises [2–4]. The use of infrared, thermal, and 3D imaging is being explored to overcome the limitations of visible modality. These modalities have shown advantages against pose, illumination, spoofing, and disguise. Infrared and thermal images are captured without visible light and are less likely to be affected by variations in illumination. Thermal sensors detect the IR radiation emitted from an object, which is converted to surface temperature. Similarly, the heat emitted by the human body as IR radiation can be recorded by thermal sensors and stored as an intensity image. In addition to robustness to illumination, thermal imagery also shows promise against disguise [5] and spoofing [6], making it suitable for security-sensitive applications of FR. These advantages of visible and thermal domains have led to an increased usage of these modalities for applications of FR.

**Figure 1:**
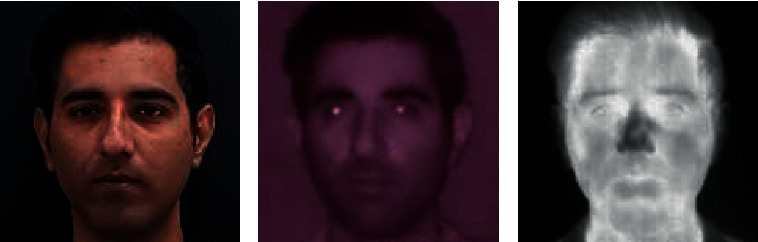
Sample images of the same subject in (a) visible, (b) infrared, and (c) thermal spectra. Visible and infrared images capture the reflection from the surface of the face while the thermal image captures the temperature of the surface.

The increasing application of different modalities for image acquisition results in an abundance of face data where the images belong to different imaging domains. This leads to scenarios where images from different domains need to be matched for face recognition. The infrared spectrum is robust to illumination, making it ideal for capturing images in the dark. This makes infrared imaging suitable for security cameras, surveillance, and monitoring around the clock. As thermal imaging captures the surface temperature of a person's face, it is a suitable choice for face recognition against spoofing and disguise. The applications of thermal imaging include border control, security-sensitive entrances, and disease monitoring for public places. As large-scale identity databases and social media platforms contain visible images only, cross-domain matching is needed to match identities across various applications and scenarios [7] such as visible images from social media and infrared images from CCTV footage. The scenario in which the modality of the probe images is different from that of the enrollment image is known as heterogeneous face recognition (HFR) [8], e.g., visible-to-infrared (VIS-NIR), visible-to-thermal (VIS-THE), and visible-to-sketch. While numerous works have been proposed for VIS-NIR [9–11] face matching, VIS-THE has received relatively less attention owing to the high cost of thermal imaging devices and the lack of availability of large-scale visible-thermal face datasets. In addition, the large modality gap between the two domains makes visible-thermal face recognition a challenging task.  Figure 1 shows the sample images for a subject in the visible, infrared, and thermal modalities. As can be observed, the VIS-NIR modality gap is significantly less prominent than the VIS-THE modality, making VIS-THE image matching a more difficult task.

Several methods have been proposed to reduce the domain gap between different modalities, e.g., common subspace projection-based methods, synthesis-based methods, and local feature extraction-based methods. However, most manually designed feature descriptors are not capable of dealing with large intraclass variations. Recently proposed deep learning–based techniques learn these descriptors automatically and have achieved promising results in multiple cross-modality scenarios. These approaches rely on the extracted embedding vector distances for training and face matching. Typically, a handcrafted loss function is utilized to project the extracted embedding vectors in a hypothetical feature space. The goal of this projection is to optimize the network to increase interclass separation and decrease intraclass distances. Furthermore, simplistic distance measures such as cosine or Euclidean distance are used at the inference stage for face matching. We hypothesize that the usage of handcrafted loss functions and hardcoded distance measures is not an optimal solution for HFR. Rather, a relational learning function that distinguishes between embeddings of different classes should be more efficient for HFR and other metric learning tasks.

Motivated by the success of convolutional neural networks for relational learning, we propose a Cross-Modality Discriminator Network (CMDN) for heterogeneous face recognition. Instead of using a distance-based loss function for face matching, the network employs a Deep Relational Discriminator (DRD) module to learn the relationships between cross-domain images. We argue that a discriminator function, learned during the network training, should outperform traditional distance metrics for HFR tasks. Furthermore, we formulate a metric learning-based loss, namely, Unit-Class Loss that is robust to a small amount of training data, noisy samples, and large modality differences in the training data. The proposed loss enhances the feature learning of the network by considering individual samples as well as the whole class distributions.

This paper presents a Cross-Modality Discriminator Network and Unit-Class Loss for heterogeneous face recognition. The proposed loss can learn modality invariant identity features from unaligned facial images. In our training process, VGGFace2 [12] weights are used to initialize the backbone network for FR. The backbone network is then fine-tuned on the IRIS [13] face database with a classification layer for visible and thermal face classification. Then, for the HFR training, the Deep Relational Discriminator module is integrated with the backbone network, and the training is performed on the HFR dataset. The proposed network can be used to (a) extract face embedding vectors, (b) obtain HFR classification, and (c) create a fusion of embedding vectors and classification probabilities. The presented network is tested on multiple datasets: the TUFTS Face database [14], Collection X1 from the University of Notre Dame (UND-X1) database [15, 16], University of Science and Technology China, Natural Visible and Infrared facial Expression (USTC-NVIE) database [17, 18], CASIA NIR-VIS [19], and Sejong Face Database [20]. Our results show that our proposed method outperforms the existing methods for VIS-THE and VIS-NIR HFR.

In this article, our main contributions are as follows:

- We propose an efficient end-to-end Cross-Modality Discriminator Network (CMND) for heterogeneous face recognition.
- We propose a Deep Relational Discriminator (DRD) module that eliminates the need for a handcrafted loss function for cross-domain face matching. Rather than using a hard-coded distance metric, the DRD module learns to differentiate between same and different class samples.
- We propose a novel Unit-Class Loss (L_uc_) for optimizing the CMND. L_uc_ compels the network to learn identity-discriminative embedding representations by penalizing intraclass variations and encouraging interclass distances.
- We demonstrate the superior performance of our proposed network over previous works on various VIS-THE and VIS-NIR face datasets.

The rest of the paper is organized as follows. In Section 2, we review the related works on VIS-NIR and VIS-THE HFR.  Section 3 presents the details of the proposed methodology. The quantitative and qualitative results on different HFR datasets are presented in Section 4. Finally, the conclusion and future works are presented in Section 5. An earlier version of this study has been presented as a preprint in arXiv [21].

## 2. Related Works

In this section, we present a review of the related literature. The significant increase in multi-modal and cross-domain data calls for methods that can meet the newly rising needs of multi-modal data [22, 23]. Usually, multi-modal domains consist of two very distinct modalities, such as images and text, or text and speech, and are restricted to the classification of close-set [24] problems. On the other hand, HFR considers very similar data from different imaging domains and need the system to be able to handle open-set verification, making it a more challenging task. A close-set problem is where the system is trained to classify categories previously seen during training, such as object recognition, whereas in an open-set scenario the system should be able to match unseen identities at the inference stage. Person re-identification [25] is a similar problem where a subject's whole body is matched against its previously stored information. While person re-identification considers all existing features of the body, such as clothes, color, height, gait, etc., HFR only considers the face features for identity matching. Heterogeneous face matching has been receiving increasing attention from the biometrics community. There are three categories of the existing methodologies for HFR, namely, latent subspace learning, cross-domain synthesis, and feature extraction-based methods.

### 2.1. Latent Subspace Learning

Latent subspace learning-based algorithms project cross-domain features to a common latent space, in which the similarity of heterogeneous information can be compared. The most common approach is to derive a set of facial features from both domains, such that the modality-related information is removed, and the identity-related information is retained. This is done by finding a mapping for both modalities to a common feature subspace. Zhu et al. [10] proposed a transductive heterogeneous face-matching method. They first apply Log-DoG filtering, local encoding, and uniform feature normalization to reduce the domain gap between VIS-NIR images. Then, a transductive classifier is trained for face matching. Yi et al. [26] extracted Gabor features at localized facial points and then used Restricted Boltzmann Machines (RBMs) to remove the heterogeneity around the focal points by learning a shared representation. Then, these shared representations of local RBMs were connected and classified using PCA. Klare et al. [27] proposed a generic HFR framework where the probe and enrollment images are represented in terms of nonlinear similarity by using a prototype random subspace (similarity kernel space) such that the prototype subjects each have an image in both modalities. Hu et al. [28] proposed to use discriminant partial least squares (PLS) by specifically building PLS gallery models for each subject with the help of thermal cross examples. The images are first preprocessed by the PLS regression-based approach to reduce the domain gap and then the recognition is performed using a one-vs-all model. He et al. [29] proposed to use a deep convolutional network approach to learn a mapping that can project both VIS and NIR images to a common compact Euclidean space. The training strategy is like that of Ref. [30]. Reale et al. [31] applied coupled dictionary learning to the thermal-to-visible matching problem. The coupled dictionaries representing the two domains provide a sparse representation that transforms the data into a single, domain-independent, latent space. Reale et al. [32] propose to use a complex deep model to learn the mapping for both VIS and NIR faces into a domain-invariant latent feature space so that they can be compared directly. Peng et al. [33] proposed to use Markov networks to extract features so that the spatial information can be preserved, and patches are extracted for both probe and gallery images. These patches are then compared using a coupled representation similarity metric. Latent subspace-based methods need to find the mapping of the input domain to a target domain using manually selected feature extractors. These manually designed extractors fail in scenarios where there are large variations in data. Deep learning–based methods offer a solution for generic feature extractors which can be trained for the specific dataset and domains.

### 2.2. Cross-Domain Synthesis

*Cross-Domain Synthesis* methods aim to generate an image in the gallery domain from the given test image, so that heterogeneous face recognition can be treated as homogenous face recognition. Typically, these methods involve two steps; first, a visible image is synthesized from a thermal image, and next, face matching is performed for the synthesized image and the gallery. Li et al. [34] proposed one of the earlier attempts using a learning-based model to synthesize visible images from thermal counterparts. Iranmesh et al. [35] proposed to use coupled generative adversarial networks (GAN) consisting of two generator and two discriminator networks to synthesize visible images from thermal images. The FR is then performed on the intermediate network features. Fu et al. [36] propose a dual variational generator elaborately designed to learn the joint distribution of paired heterogeneous images. The variational generator is then used to increase the multi-modal training data by synthesizing infrared images from visible images. The increased data is then used to train an HFR network. Synthesis-based methods rely on GANs which lack an objective function, making it difficult to evaluate the quality and validity of the generated data [37].

### 2.3. Modality Independent Feature Learning

Modality independent feature learning aims to extract features related to face identity, discarding the modality information. Various deep learning approaches have been proposed for cross-domain FR. Ghosh et al. [38] proposed a subclass heterogeneity-aware loss to train deep neural networks for cross-domain and cross-resolution face recognition. Deep perceptual Mapping [39] captures the highly nonlinear relationship between the visible and thermal modalities by using a deep neural network. A deep neural network is used which attempts to learn a nonlinear mapping from visible to thermal spectrum while preserving the identity information. Riggan et al. [40] used coupled auto-associative neural networks along with deep perceptual mapping (DPM) to learn common features that are useful for cross-domain face recognition. He et al. [30] proposed Wasserstein Convolutional Neural Network to learn domain-invariant features. The low-level layers are trained using the VIS images, and the high-level images are further divided into three parts, i.e., NIR layer, VIS layer, and NIR-VIS shared layer. The first two layers aim to learn modality-specific features, whereas the shared layer aims to learn modality-invariant features.

Metric learning losses and their variations have been used for face verification, but these are sensitive to the selection of image pairs and require hard-sample mining for effective training. Synthesis-based methods rely on effective training of generative adversarial networks, which is an inherently complex problem. Facial feature–based methods depend on facial alignment, which is an independent problem for highly noncorrelated modalities. Various effective approaches have been proposed using multiple network branches, but this increases the network parameters multifold and requires a large number of training resources. This paper overcomes the shortcomings of the current methods by proposing an end-to-end deep relational network for cross-domain face matching.

## 3. Proposed Method

**Figure 2:**
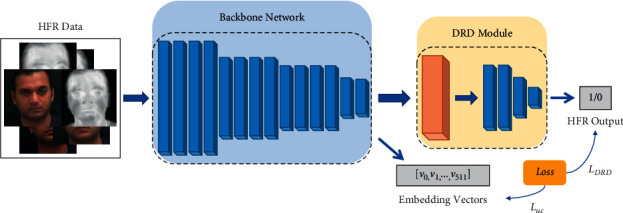
The architecture of the proposed cross-modality discriminator network. The network has two outputs, a vector embedding representing modality independent identity and a HFR match score.

This section presents the building blocks of our proposed algorithm. The goal of an efficient cross-modality recognition system is to project the input images such that the intraclass projections have a small distance whereas interclass projections have a large distance. The choice of our backbone architecture, the motivation for our loss function, and the details of the Deep Relational Discriminator module for achieving this goal are explained below.  Figure 2 shows the overall architecture of the proposed network. For a given cross-modality image pair, the network outputs an embedding vector for each image and a match HFR score. The embedding represents the identity of the face in the image as a 256 × 1 float vector. These embeddings can be compared using cosine distance to find similar identities in the gallery set. The HFR output by the network is a float value between 0 and 1, where a higher value signifies high similarity between the input image pair.

### 3.1. Backbone Architecture

Deep residual networks were introduced to solve the issue of degradation in deep neural networks [41]. They introduced skip connections with the identity function to allow the gradient of the cost function to progress directly from deeper layers to shallow layers. The identity blocks are also effective at improving the performance of networks when vanishing gradients and degradation are not an issue. The efficiency of deep residual networks (ResNet-50) [41] has been proven for face recognition on the MS1M [42] and VGGFace2 [12] datasets.

Squeeze and Excitation (SE) blocks recalibrate channel-wise feature responses by explicitly modeling the interdependencies between channels. The SE block models channel interdependencies by selectively emphasizing informative channels and suppressing less useful ones. For the transformation *x*_*l*_ to *x*_*l*+1_, squeeze (F_sq_), excitation (F_ex_), and scaling (F_sc_) operations are performed for a given feature matrix *x*_*l*_ of size H × W × C. The advantages of integrating SE blocks have been demonstrated for face recognition using the VGGFace2 dataset. In this work, we use the ResNet-50 with SE blocks (SENet-50) [43], architecture as our backbone network. The backbone network takes a 224 × 224-sized image input and calculates a 256-dimensional embedding vector of the input image.

### 3.2. Triplet Loss

**Figure 3:**
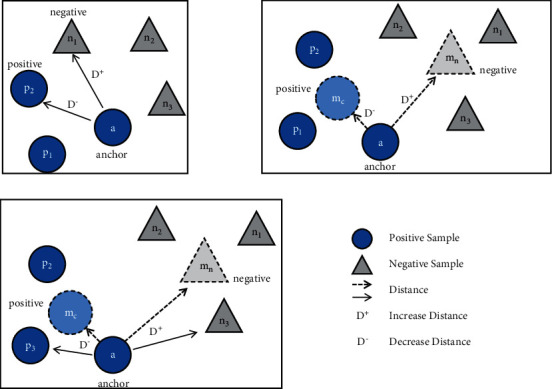
The calculation of loss functions (a) triplet loss, (b) class mean triplet loss, and (c) unit-class loss. The triplet loss considers the embedding distances between an anchor, a positive class image, *p*_2_, and negative class, *n*_1_. The Class mean triplet loss considers the distances between an anchor, a positive class mean, *m*_c_, and negative class mean, *m*_n_. The Unit-Class Loss considers the distances between an anchor, a positive class mean, *m*_c_, negative class mean, *m*_n_, positive class image, *p*_3_, and negative class, *n*_3_.

Triplet Loss was used by Schroff et al. [44] for face recognition and clustering. Since then, triplet loss and its variations have been used for single-modal, multi-modal, and cross-modal face recognition. For the extracted embedding vector of a given anchor image, positive image, and negative image, the triplet loss aims to minimize anchor-to-positive distance and increase anchor-to-negative distance. The idea is to train the network such that the same-class images are projected in the nearby region, whereas nonclass images are projected farther from the anchor.

(1) $$\begin{array}{c} L_{T}\left(a,p,n\right)=Max\left(0,D\left(a,p\right)-D\left(a,n\right)+\alpha \right)\\ a=\Theta \left(I_{a}\right), \end{array}$$

where Θ(*·*) is the feature extraction function of the network, *I*_*a*_ is the anchor image, *a* is the vector embedding for image *I*_*a*_, and *L*_*T*_(*a*, *p*, *n*) is the loss function for the embedding vectors *a*, positive class image *p*, and negative class image *n*. *D*(*·*, *·*) is the distance function for the learned vector representations of two images. *L*2 or cosine distances are typically used as distance measures and *α* is the margin parameter usually set to 1.0. A graphical illustration of the triplet loss is shown in Figure 3(a).

### 3.3. Class Mean Triplet Loss

The goal of a heterogeneous face recognition system is twofold: (a) to minimize the distances between multi-modal image representations of the same class and (b) to increase the distance between image representations of different classes. Given a vector representation *a*_*c*_, of an image of class *c*, the class mean triplet loss is defined as follows:

(2) $$\begin{array}{c} L_{S}\left(a_{c},m_{c},m_{n}\right)=Max\left(0,D\left(a_{c},m_{c}\right)-D\left(a_{c},m_{n}\right)+\alpha \right), \end{array}$$

where

(3) $$\begin{array}{c} m_{c}=\frac{1}{I_{c}}\sum_{k=1}^{I_{c}}\Theta \left(x_{k}\right),\\ m_{n}=\frac{1}{I_{n}}\sum_{k=1}^{I_{n}}\Theta \left(x_{k}\right),\\ n\in \left\{U-c\right\}. \end{array}$$

Here *U* is the universal set containing the classes. *L*_*S*_(*a*_*c*_, *m*_*c*_, *m*_*n*_) is the loss function for the vector representations of the anchor image *a*_*c*_, belonging to class *c*. *m*_*c*_ and *m*_*n*_ are the means of vector representations in classes *c* and *n*, respectively. Figure 3(b) shows the conceptual representation of the class mean triplet loss.

### 3.4. Unit-Class Loss

The training procedure for triplet loss is sensitive to the selection of effective samples, noise, outliers, number of classes, and minibatch diversity. Using class means for loss calculation is more robust to noise and random sample selection. However, convergence becomes more difficult because of the larger number of parameters involved. We present a novel Unit-Class Loss (L_uc_) that combines principals from triplet loss and class mean triplet loss to benefit from the advantages of both, as shown in Figure 3(c). The weight parameter *β* is introduced for the optimal weight distribution of sample-based and class-mean-based optimization of gradients. The Unit-Class loss is formalized as

(4) $$\begin{array}{c} {\text{L}}_{\text{uc}}=\text{Max}\left(0,\left(1–\beta \right)\left(D\left(a_{c},m_{c}\right)-D\left(a_{c},m_{n}\right)\right)+\beta \left(D\left(a,p\right)-D\left(a,n\right)\right)+\alpha \right).\\ \; \end{array}$$

It is to be noted that L_uc_ is the weighted sum of triplet loss and class mean triplet loss. This means that there is no computational or memory advantage offered for the calculation of L_uc_. However, the proposed loss function optimizes the network faster, reducing the required network training cycles (epochs) and consequently improving the memory and time consumption of the training process.

### 3.5. Deep Relational Discriminator Module

We hypothesize that a network trained with the end goal of matching cross-modal face pairs would train more efficiently than a network trained to project class vector representations. Traditionally, vector distance measures such as Euclidean or cosine distance have been employed to calculate the similarity between the vector representations of two images. We introduce a Deep Relational Discriminator (DRD) module to distinguish same-class face images from different class face images.

**Figure 4:**
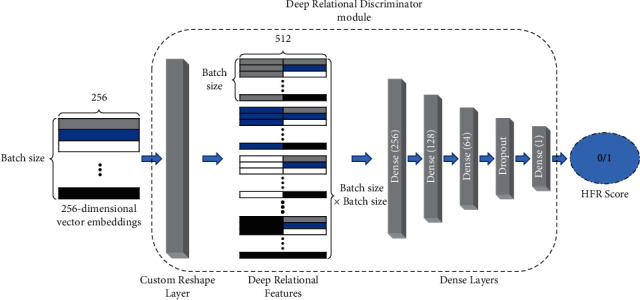
The architecture of the deep relational discriminator module. The 256-dimensional vector embeddings are extracted by the backbone network and concatenated by the custom reshape layer. The concatenated deep relational features are then used to train the subsequent layers for HFR.

An *L*2-normalized 256-dimension embedding vector of the input image is obtained from the backbone network and processed such that each image embedding is concatenated with every image. The *b* × 256 output, where *b* is the batch size and 256 is the embedding dimension, is remapped to the *b* × *b* × 512 (batch size  ×  batch size  ×  vector size) dimension input for the DRD module.

(5) $$\begin{array}{c} \text{DRD input}=a_{i}\left\|\right)a_{j,} \end{array}$$

where *i*, *j* ∈ *U*, *a*_*i*_, and *a*_*j*_ are the vector representations of images *i* and *j*, respectively, and *b* is the total number of images in the batch. The goal is to train the DRD module to classify the same-class images from concatenated image embeddings of an image pair. These concatenated image pair representations are then fed into subsequent dense layers followed by the sigmoid activation layer, as shown in Figure 4. The proposed DRD module is trained using binary cross-entropy loss to classify the matching image pairs. Our experiments demonstrate that the proposed module improves the network's performance and outperforms hard-coded distance measures for face matching.

As the DRD module outputs a match ranking between 0 and 1. We use the binary cross-entropy loss defined as,

(6) $$\begin{array}{c} L_{\text{DRD}}=-\frac{1}{n^{2}/2}\sum_{a,b}^{n,n}y\left(I^{D_{1}},I^{D_{2}}\right)\cdot \;\log\left(\;p\left(a,b\right)\right)+\left(1-y\left(a,b\right)\right)\cdot \;\log\left(1-p\left(a,b\right)\right). \end{array}$$

Here, *n* is the total number of images in a batch, *n*^2^ is the total number of image pairs, *a* ∈ {1,…, *n*} and *b* ∈ {1,…, *n*}, and

(7) $$\begin{array}{c} y\left(a,\;b\right)=\left\{\begin{array}{c} 1,\text{ for same identity}I^{D_{1}}\text{and}I^{D_{2}}\\ 0,\text{for different identity}I^{D_{1}}\text{and}I^{D_{2}} \end{array}.\right) \end{array}$$

### 3.6. Training Process

**Figure 5:**
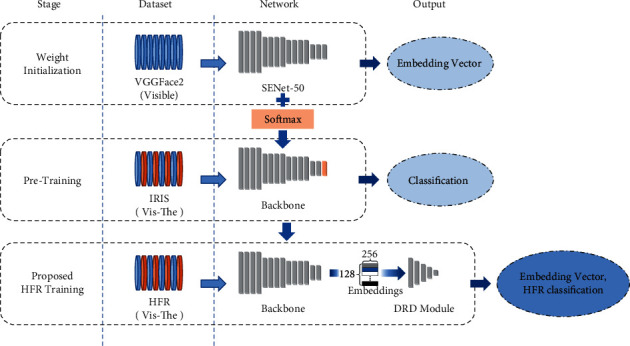
Overview of the proposed methodology and training pipeline. First, the backbone network is trained for weight initialization on the visible face dataset. Then, the network learns to classify visible and thermal faces from the IRIS face dataset. In the final stage, we integrate the proposed Deep Relation Discriminator module and train for HFR on the required dataset.

This section presents the training algorithm for the proposed method in detail. We aim to reduce intraclass variations across the domains while increasing interclass distances. Furthermore, the proposed DRD module improves the network's ability to differentiate between same-class and different-class embedding vectors. The proposed modules and loss are generic and can be integrated with the existing state-of-the-art architectures for a further boost in performance.  Figure 5 shows an overview of the proposed training methodology.

Our training process consists of three stages: weight initialization, pretraining, and HFR training. Network weights from a SENet-50 trained on the VGGFace2 [12] face database are used to initialize the backbone network. As the VGGFace2 dataset contains only visible images, we pretrain the base network on a VIS-THE face dataset to learn thermal as well as visible feature extraction. The IRIS [13] face dataset is used to pretrain the network using cross-entropy loss. The dataset contains 2,552 images each in visible and thermal modalities for 29 subjects. The facial images contain variations in expression, pose, and illumination. Feature-wise centering and feature-wise standard normalization calculated on the entire dataset (visible and thermal images) are applied to each sample. Furthermore, geometric transformations are used as data augmentation techniques to mitigate overtraining. The training data is fed in a pseudo-random manner, ensuring a randomized class and modality distribution. The network is trained until there is no further decrease in loss. We choose cross-entropy loss to train the network at this stage, given its established performance and stable nature for FR algorithms.

For HFR training, the last Softmax layer from the pretraining stage is replaced with an *L*2-normalized dense layer, and the network is connected to the proposed DRD module. The resulting network contains two outputs, i.e., the *L*2-normalized embedding vectors and the binary classification from the DRD module. We optimize the network using the proposed *L*_*uc*_ and *L*_*DR*  *D*_ for vector representations and DRD output, respectively. Class identities are passed as label information to Unit-Class Loss and the DRD Loss. The pretraining of the backbone network and the training process of the proposed network are summarized in Algorithms 1 and 2, respectively. It should be noted that our training and testing procedure does not require specific image preprocessing, facial alignment, or landmark labeling for training or testing.

## 4. Experimental Evaluation

In this section, we evaluate the performance of the proposed architecture on popular VIS-THE and VIS-NIR face datasets. We provide comprehensive parameter settings and implementation details for research reproducibility. Rank-1 accuracy and verification rates (VR) at False Acceptance Rate (FAR) of 1% and 0.1% are compared to previously proposed HFR algorithms.

### 4.1. Databases

**Figure 6:**
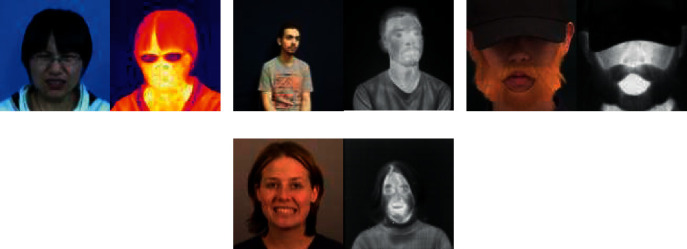
Sample visible (left) and thermal (right) images from (a) USTC-NVIE, (b) TUFTS, (c) Sejong face database, and (d) UND-X1 database.

The experiments were performed on USTC-NVIE [17, 18], TUFTS [14], UND-X1 [15, 16], and Sejong Face Database [20] for VIS-THE and CASIA NIR-VIS 2.0 face database [19] for VIS-NIR HFR.  Figure 6 shows the sample images from the HFR database.

The USTC-NVIE database [17, 18] is a facial expression database with visible-thermal image pairs for 215 subjects. The database is further divided into two subsets: a spontaneous database consisting of image sequences from onset to the apex of facial expressions and a posed database consisting of apex images. The images are captured with three illumination variations, i.e., illumination from left, right, and front. This is an expression recognition database but is used for VIS-THE HFR in this study. Among the 215 subjects, 126 subjects were found to have usable data (having sufficient images in both modalities). Both sub-datasets are used to maximize the number of training and test data. The training was performed using 1600^2^ image pairs (visible and thermal images) from 100 subjects. 416^2^ image pairs from 26 subjects were used for testing.

The TUFTS database [14] contains data belonging to multiple categories, i.e., 2D visible, thermal, Infrared, 3D, 3D LYTRO, Sketch, and Video. The database contains images having variations in pose, expression, and sunglasses. To avoid the additional challenge of pose, only frontal images with variations in expression and glasses were used for training and testing. Thermal and visible image corpus was used for our HFR experiments. The training was performed using 481^2^ image pairs (visible and thermal images) of 74 subjects. Here, 247^2^ image pairs of 38 subjects were used for testing.

UND-X1 [15, 16] contains 82 subjects with LWIR and visible light image pairs with varying illumination, expression, and time-lapse. Out of 82 subjects, 50 subjects' images were used for training. Forty image pairs were used for each subject. The training was performed using 1000^2^ image pairs from 50 subjects. The test was performed using 1280^2^ image pairs of 32 subjects.

Sejong Face Database [20] (SFD) contains images in visible, infrared, and thermal modalities for 100 subjects. The subjects are captured wearing disguises and facial add-ons such as fake beards, caps, scarves, and wigs, with and without makeup, and so on. The addition of facial add-ons makes HFR a more challenging problem for SFD. 975^2^ image pairs from 75 subjects were used for training and 325^2^ image pairs from 25 subjects were used for testing.

The CASIA NIR-VIS 2.0 [19] database contains visible-infrared image pairs for 725 subjects. The number of images for the subjects ranges between 1–22 and 5–50 for visible and infrared, respectively. The database contains two views of the evaluation protocols. View-1 is used for training and View-2 is used for testing. To simulate the practical situations, the VIS images are used as a gallery and the NIR images are used as the probe. For each subject in the gallery set, only one VIS image is selected. For a fair comparison with other results, we follow the standard protocol in View-2 for testing.

### 4.2. Training Parameters and Implementation Details

The proposed network is implemented using TensorFlow [45], an open-source deep learning framework. The experiments are performed using two Nvidia GTX 1080Ti GPUs. In the pretraining stage, we adopt the IRIS Visible-Thermal image dataset to train the backbone network. At this stage, the categorical cross-entropy loss is used for multi-modal face recognition. Here, 5,100 images for 29 subjects are used as training data. The training images are resized to a size of 224 × 224 pixels and the labels contain class information only. The network is trained using an Adam optimizer [46] with an initial learning rate of 3*e*^−4^ and the learning rate is reduced by a factor of 0.8 when the error plateaus. The network is trained using a minibatch size of 64 (32 visible and 32 thermal images) until the loss plateaus.

In the training stage, the Softmax layer is replaced with a 256-dimension *L*2-normalized dense layer and the DRD module is added to the head of the network. The backbone network is trained on the HFR dataset with the proposed Unit-Class Loss and the DRD head is trained using cross-entropy loss. The values of *α* and *β* are set to 1.6 and 0.6, respectively. The batch size is set to 128 (64 visible and 64 thermal) images. The Adam optimizer, with an initial learning rate of 3*e*^−4^, is used for gradient descent optimization. The learning rate is reduced by a factor of 0.8 when the loss plateaus. The fine-tuning process for each dataset takes approximately 2 hours.

Owing to the difference in image sizes between different modalities and across different databases, the images are cropped to match the shorter edge. The square images are then scaled to 224 × 224 pixels for all databases. Data augmentation is performed to mitigate overtraining and increase training size. Geometric transformations for rotation shift in both axes, brightness shift, shear, and horizontal flip are applied to the training data. Image normalization is performed using feature-wise centering and feature-wise standard normalization calculated on the entire training dataset.

### 4.3. Training and Test Data

To verify the performance of our proposed HFR algorithm, we compare our method with the state-of-the-art HFR methods. The number of available test subjects, gallery images, and probe images for the face verification experiments is listed in Table 1.

The proposed network can be used in multiple ways: (a) the embedding vectors (*emb*) can be retrieved from the *L*2-normalized layer for a single image and HFR performed using the cosine distance between the gallery and probe images, (b) an HFR classification (*hfr*), i.e., genuine versus imposter image pair, for an image pair can be extracted from the DRD output, (c) score fusion (*fus*) for distance measure of *emb* and *hfr* output can be performed to achieve an average recognition measure. The Rank-1 accuracy and for *emb*, *hfr*, and *fus* are presented. The *emb* results are computed for one visible image matched against the whole thermal gallery. For testing the *hfr* results, all available VIS-THE image pairs from the test set are used. *Fus* score is reported on the test pairs used for embedding recognition.

### 4.4. Evaluation Metrics

Given two test images, the 1 : 1 verification determines if the images belong to the same identity. For the embedding output of the model, the distances are calculated using the cosine distance between the extracted embedding features and the same or different identity classification performed based on a threshold value. For the *hfr* output of the model, the model outputs the same identity probability for the input image pair. 1 : N Identification determines the matching identity from a gallery of N images for a given probe image. We present Rank-1 recognition rate and true positive rate (TPR) at False Acceptance Rate (FAR) of 1% and 0.1% for face recognition and verification tasks [47]. Rank-1 accuracy is defined as the proportion of correctly predicted face image pairs (True positive) among the total number of image pairs,

(8) $$\begin{array}{c} \text{Accuracy}=\frac{TP+TN}{TP+TN+FP+FN}, \end{array}$$

where TP = True positive, FP = False Positive, and FN = False Negative. TPR at a FAR is described as the number of correct predictions at a specific percentage of acceptable false predictions. TPR and FAR are calculated as

(9) $$\begin{array}{c} \text{TPR}=\frac{TP}{\left(TP+FN\right)},\\ \text{FAR}=\frac{FP}{\left(FP+TN\right)}, \end{array}$$

The threshold is defined to determine the FAR.

### 4.5. Experimental Results

Table 2 presents the Rank-1 identification and verification rates on UND-X1. We compare the performance of our method with those of the recent CpGAN [35], DPM [39], DVG-Face [36], and W-CNN [30]. As can be seen in Table 2, the proposed network achieves Rank-1 accuracy of 95.21% with the *fus* output, which is a significant improvement over 83.73% and 76.45% recognition rate achieved by DPM and CpGAN, respectively. While DPM and CpGaN aim to find a translation from thermal to visible images, the proposed method focuses on identity feature extraction and learning the relationships between those features. DPM achieves similar performance in terms of verification rate compared to the proposed *emb* and *hfr*, but the proposed method outperforms CpGAN by a large margin. Overall, the proposed method achieves an improved performance on the UND-X1 dataset.

We compare our results with the existing results on the TUFTS dataset. As the TUFTS database is relatively new, few VIS-THE HFR results have been reported. DVG-Face [36] proposes a two-step dual variation generation method to generate thermal images from visible images. The generated dataset is used to train a LightCNN for recognition. As can be seen in Table 3, we achieve a marginally improved Rank-1 recognition rate over DVG-Face for *emb* output, but the proposed *hfr* and *fus* achieve a significant improvement of 21.3% and 22.8% over the current methods, respectively. The proposed end-to-end CMDN outperforms the two-stage methods by minimizing error. Further baseline results using triplet loss for the TUFTS database are presented in ablation studies.

As USTC-NVIE is primarily an expression database; it lacks significant HFR results. We report baseline results for triplet loss in ablation studies. The PCA, Fisherface, and G-HFR results are presented from a previous study [33] in Table 4. The proposed *fus* method achieves a Rank-1 recognition rate of 99.7% compared to G-HFR, showing that the proposed method outperforms graphical representations of facial identities as proposed by G-HFR. Furthermore, the proposed *emb* and *hfr* achieve very similar recognition rates to *fus*, of 99.3% and 99.4%, respectively. The proposed *emb* achieves the highest TPR@FAR for the NVIE dataset, closely followed by *hfr* and *fus*.

The Sejong Face Database has been proposed for disguised face recognition across various modalities. The inclusion of facial add-ons that hide large parts of the face makes it a particularly challenging face dataset. The Rank-1 recognition rates for single-modal visible images (single-modal) and multi-modal images (visible, thermal, and infrared) using score fusion (score-fusion) on the database are reported using the methods reported in Ref. [20]. Furthermore, the database is tested for VIS-THE HFR in detail using DPM, CpGAN, DVG-Face, IDNet [32], and IDR [29], and the results are reported in Table 5. As can be observed, the HFR recognition rates for W-CNN (74.9%) and IDR (74.3%) are similar to single-modal recognition (77.2%). As the single-modal FR is a simpler problem, better results are expected for FR compared to HFR. Using multiple modalities (score-fusion) improves (92.3%) the face recognition results as multi-network ensemble and additional image data are being used for recognition. The proposed method, with a single network and for cross-domain matching achieves a Rank-1 recognition rate of 92.4%, a significant improvement over other methods.

CASIA NIR-VIS 2.0 is used to present our results on the VIS-NIR modality. The dataset has two subsets; View-1 is used for training and View-2, with 10 different splits, is used for testing. The CASIA NIR-VIS 2.0 restricts the testing to one gallery image per subject. Therefore, there are 358 visible gallery images and about 6000 probe infrared images for testing.  Table 6 Summarizes the results of our proposed model compared to other recent methods applied on CASIA NIR-VIS 2.0. It should be noted that the proposed network is designed and optimized to perform on VIS-THE data. The proposed method achieves a Rank-1 recognition rate of 99.5%, compared to 98.7% achieved by W-CNN and 97.3% achieved by the IDR method. While the proposed method achieved the highest TPR@FAR of 1%, IDR performs better for TPR@FPR of 0.1%.

### 4.6. Cause Analysis

The proposed network offers multiple advantages over the currently proposed methods for HFR. The Cross-Modality Discriminator Network employs a single backbone, which learns shared weights for both modalities. The usage of shared weights prevents overtraining of the network and results in better open-set performance. Moreover, having a single network, trained for end-to-end HFR avoids the possible loss accumulation (as opposed to multiple networks), resulting in improved recognition rates. Instead of using a hard-coded loss function, the proposed DRD module learns the interclass and intraclass relationships for deep features of test images. The learned relationships used for HFR classification outperform the manually designed feature descriptors as shown by the performance of the proposed method. Finally, the proposed CMDN gives two outputs, embedding vectors, and the match probability for an image pair, which are combined to improve HFR performance over individual outputs.

### 4.7. Ablation Studies

To verify the effectiveness of our proposed method, we perform ablation studies to explore the effects of different loss functions, the DRD module, and different values of *α* and *β*. The Rank-1 accuracy and VR for the USTC face database are reported in Table 7. The results are calculated using embedding distances for a fair comparison. The ablation experiments are performed as follows:

1. Experiment 1: The SENet-50 backbone network is trained using triplet loss without the DRD module
2. Experiment 2: The SENet-50 backbone network is trained using the class mean triplet loss without the DRD module
3. Experiment 3: The network is trained using the proposed class mean triplet loss without the DRD module
4. Experiment 4: The network is trained using the proposed Unit-Class Loss without the DRD module
5. Experiment 5: The network is trained using triplet loss and the DRD module is added
6. Experiment 6: The network is trained using the proposed Unit-Class Loss and the DRD module (proposed method)

As can be seen in Table 7, the combination of Unit-Class loss and DRD module outperforms the ablation variations in terms of Rank-1 accuracy and verification rate. The addition of the DRD module with triplet loss achieves the second-best performance as the network is reinforced for the final goal of HFR as well as embedding vector optimization. The effects of changing the values of *α* and *β* are shown in Table 8. As can be seen, changing the value of *β* affects the network performance, whereas changing the value of *α* does not have a noticeable effect on performance. We determine the values *α*=1 and *β*=0.5 to be optimal for our HFR results.

**Figure 7:**
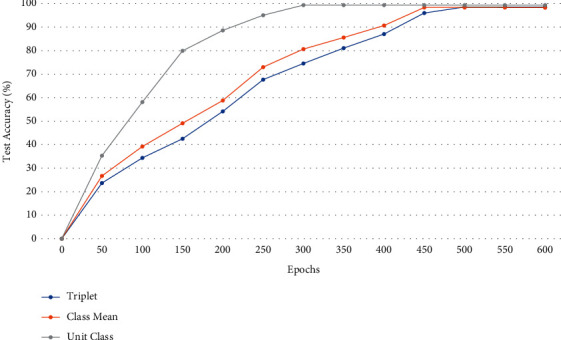
The test performance of the proposed network trained different loss functions.

Figure 7 shows the test accuracy of the proposed network trained with the triplet loss, class mean triplet loss, and unit-class loss for incremental training cycles. As can be seen, the network trained with unit-class loss achieves improved performance over the networks trained with triplet loss and class mean triplet loss starting from 50 training epochs. The optimal performance is achieved using the proposed loss function in 300 epochs, while the networks trained with triplet and class mean triplet loss achieve their optimal performance after 500 and 450 epochs, respectively. This shows that the proposed loss function not only improves network performance but also decreases memory and time consumption of the training process by reducing the required training epochs.

## 5. Conclusion

We present an end-to-end network for visible-to-thermal HFR using a novel Unit-Class Loss and a Deep Relational Discriminator module. The backbone network is initialized with the weights trained on a large-scale visible dataset. Next, the multi-modal features are learned through training on a visible-thermal database using cross-entropy loss. The backbone network is then integrated with the proposed Unit-Class Loss and DRD module for HFR. The proposed loss function maximizes positive-to-negative pair distance by reducing intraclass variations and increasing interclass variance. The HFR performance is further enhanced by deep relational learning to classify same class image pairs. The experiments on multiple cross-domain face datasets prove that the proposed CMDN outperforms the existing state-of-the-art methods on visible-thermal and visible-infrared datasets, validating the effectiveness of the proposed method.

The usage of the proposed loss and discriminator module is simple and can be adapted to any network architecture for other HFR modalities. Furthermore, the methodology can be adapted for generic processing machines given its low computational complexity, making further research and industrial application feasible. In the future, more advanced architectures can be adapted to further improve and fine-tune the proposed strategy for other heterogeneous problems. Incorporating modality labels into training is also worth exploring. In the future, we will explore optimizing our network for other heterogeneous recognition problems.

## Figures and Tables

**Algorithm 1:**
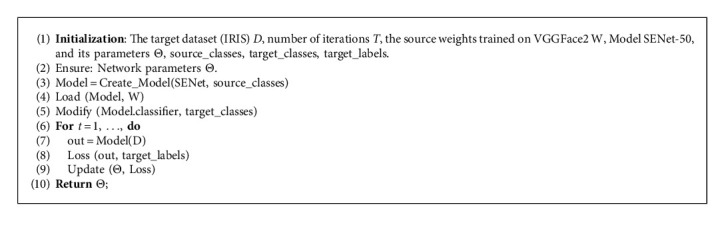
Pretraining the backbone network.

**Algorithm 2:**
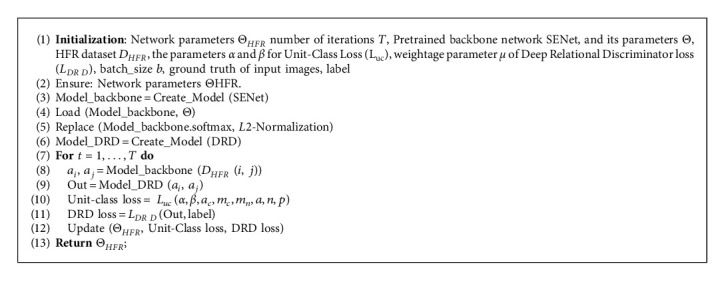
Training the proposed network on HFR data.

**Table 1:** The number of available test subjects, training images, and test images for each subject. As the number of test and training images varies in CASIA NIR-VIS 2.0, the details are given in the database description.

Database	Train subjects	Train image pairs	Test subjects	Test image pairs
UND-X1	50	1000 × 1000	32	1280 × 1280
TUFTS	74	481 × 481	38	247 × 247
NVIE	100	1600 × 1600	26	416 × 416
SFD	75	975 × 975	25	325 × 325
CASIA NIR-VIS 2.0	357	—	358	—

**Table 2:** Rank-1 accuracy and verification rate on UND-X1 database.

Method	Rank-1 (%)	TPR@FAR = 1%	TPR@FAR = 0.1%
DVG-face	79.63	89.4	57.2
W-CNN	84.91	90.6	51.2
DPM	83.73	**91.1**	63.3
CpGAN	76.45	84.5	58.3
Proposed *emb*	80.82	90.8	52.9
Proposed *hfr*	93.63	87.7	**64.8**
Proposed *fus*	**95.21**	88.3	61.1

The bold values represent the highest value achieved in a column.

**Table 3:** Rank-1 accuracy and verification rate on TUFTS database.

Method	Rank-1 (%)	TPR@FAR = 1%	TPR@FAR = 0.1%
DVG-face	75.7	68.5	36.5
Proposed *emb*	79.2	94.7	45.6
Proposed *hfr*	97.0	**95.7**	48.0
Proposed *fus*	**98.5**	95.3	**49.1**

The bold values represent the highest value achieved in a column.

**Table 4:** Rank-1 accuracy and verification rate on USTC-NVIE database.

Method	Rank-1 (%)	TPR@FAR = 1%	TPR@FAR = 0.1%
PCA	0	0	0
Fisherface	8.72	1.3	0.5
G-HFR	77.4	89.6	61.3
Proposed *emb*	99.4	**98.4**	**71.0**
Proposed *hfr*	99.3	96.5	69.9
Proposed *fus*	**99.7**	97.4	70.3

The bold values represent the highest value achieved in a column.

**Table 5:** Rank-1 accuracy and verification rate on Sejong Face database.

Method	Rank-1 (%)	TPR@FAR = 1%	TPR@FAR = 0.1%
DPM	55.6	44.2	25.6
CpGAN	54.2	53.6	38.6
DVG-face	59.6	59.4	37.2
IDNet	65.3	55.8	35.3
IDR	74.3	45.2	36.8
W-CNN	74.9	59.6	41.2
Single-modal	77.2	—	—
Score-fusion	92.3	—	—
Triplet loss	60.4	63.5	59.6
Proposed *emb*	73.2	74.5	32.4
Proposed *hfr*	91.6	**87.7**	**41.9**
Proposed *fus*	**92.4**	85.4	40.9

The bold values represent the highest value achieved in a column.

**Table 6:** Rank-1 accuracy and verification rate on CASIA NIR-VIS database.

Method	Rank-1 (%)	TPR@FAR = 1%	TPR@FAR = 0.1%
IDNet	87.1	98.5	74.5
IDR	97.3	98.9	**95.7**
W-CNN	98.7	99.5	98.4
Proposed *emb*	97.5	**99.8**	78.2
Proposed *hfr*	99.2	99.5	78.6
Proposed *fus*	**99.5**	99.3	77.8

The bold values represent the highest value achieved in a column.

**Table 7:** Rank-1 accuracy and verification rate for the ablation studies on the USTC database.

Method	Rank-1 (%)	TPR@FAR = 1%	TPR@FAR = 0.1%
Exp. 1: triplet loss	95.6	86.1	43.1
Exp. 2: class mean loss	97.8	91.2	53.8
Exp. 3: class mean loss + DRD module	98.4	93.4	64.2
Exp. 4: unit-class loss	97.9	88.4	56.2
Exp. 5: triplet loss + DRD module	98.6	96.1	67.1
Exp. 6: proposed	**99.4**	**98.4**	**71.0**

The bold values represent the highest value achieved in a column.

**Table 8:** The Rank-1 recognition rate of the proposed CMDN with different values of *α* and *β*.

*α*	*β*	Rank-1 (%)
0.5	0.2	98.7
1	0.2	98.5
2	0.2	98.6
0.5	0.5	99.4
1	0.5	**99.4**
2	0.5	99.3
0.5	0.8	97.9
1	0.8	97.9
2	0.8	98.9

The bold values represent the highest value achieved in a column.

## Data Availability

Previously reported face image databases were used to support this study and are available at their respective repositories. These prior datasets are cited at relevant places within the text as references for USTC-NVIE [17, 18], TUFTS [14], UND-X1 [15, 16], Sejong Face Database [20], and CASIA NIR-VIS 2.0 face database [19]. The source code and data used to support the findings of this study have been deposited in the GitHub repository (https://github.com/usmancheema89/ForkCNN-Triplet).
